# A Review of Distribution and Profiles of HBCD in Different Environmental Media of China

**DOI:** 10.3390/molecules29010036

**Published:** 2023-12-20

**Authors:** Jinglin Deng, Wenbin Liu, Lirong Gao, Tianqi Jia, Yunchen He, Tianao Mao, Javid Hussain

**Affiliations:** 1 Research Center for Eco-Environmental Sciences, Beijing 100085, China; dengjlin@yeah.net (J.D.); gaolr@rcees.ac.cn (L.G.); tqijia@126.com (T.J.); yunchenhe@yeah.net (Y.H.); 2 College of Resources and Environment, University of Chinese Academy of Sciences, Beijing 101408, China; maotianao@foxmail.com (T.M.); javid.hussain@buitms.edu.pk (J.H.); 3 Hangzhou Institute for Advanced Study, University of Chinese Academy of Sciences, Hangzhou 310024, China; 4 Department of Environmental Sciences, Balochistan University of Information Technology, Engineering and Management Sciences, Quetta 87100, Pakistan

**Keywords:** HBCD, occurrence and distribution, environmental media

## Abstract

Hexabromocyclododecane (HBCD) is the most important flame retardant that has been used in Expanded Polystyrene foam and Extruded Polystyrene foam in the past forty years across the world. China was the major producer and user of HBCD, and the total HBCD production was about 0.3 million tons. Although HBCD was completely banned in China in 2021 because of its long-range transport, bioaccumulation and toxicity, there is still a lot of residue in the environment. Therefore, we reviewed multiple studies concerning the distribution of HBCD in diverse environmental matrices, such as in the air, dust, soil, water, sediment, and biota. Results revealed that HBCD levels in different environments in China present geographical variation and were at a high level compared with other countries. In all environmental media, relatively high HBCD concentrations have been found in industrial and urban areas. Industrialization and urbanization are two important factors that influence the concentration and distribution of HBCD in the environment. In terms of isomer, γ-HBCD was the dominant isomer in soil, water, and sediment, while in the biota α-HBCD was the predominant isomer.

## 1. Introduction

Hexabromocyclododecane (HBCD, C_12_H_18_Br_6_) was a class of persistent organic pollutants with obvious toxicity (carcinogenicity and mutagenicity), which was commonly found in a wide range of environmental media, including the atmosphere, water, and organisms. HBCD in the environment was characterized by difficult degradation, long-range transport, bioaccumulation, and low water solubility [1,2,3,4,5,6]. The possible adverse effects of exposure to HBCD were of public concern due to its highly toxic properties. And, HBCD was listed in the Stockholm Convention on Persistent Organic Pollutants in 2013.

HBCD has been mainly applied as a flame retardant in Expanded Polystyrene foam (EPS) and Extruded Polystyrene foam (XPS) for insulation and construction [7]. Among the brominated flame retardants (BFRs) used worldwide, HBCD has the third highest production volume. China was the major producer and user of HBCD [8]. According to our joint investigation with the relevant associations, the total production of HBCD was about 300,000 tons, in addition to 26 million tons of EPS and 2.3 million tons of XPS containing HBCD in China. The production and use of HBCD has been an increasing trend due to improvements of fire awareness and safety standards in China. Between 2001 and 2009, the annual production of HBCD doubled approximately 20 times to 10,000 tons [9]. After 2010, annual production of HBCD jumped to 20,000 tons and then remained constant at approximately 18,000 tons until 2021. Although the production and use of HBCD was completely banned in China in 2021, the products and wastes containing HBCD can still enter and affect the environment due to its slow release. The residual HBCD in the environment is another potential pollution source of HBCD. 

As a signatory to the Stockholm convention, China has made a lot of effort in HBCD-pollution prevention and control, and many scientists in China have investigated the occurrence, emission, and environmental pollution of HBCD from various sources. More studies were conducted around 2015, with a downward trend in studies after 2019. However, the latest overall contamination levels of HBCD in different environmental media at the national level since 2021 have not been summarized and reported. We reviewed over 100 studies on the distribution of HBCD in China during the last 20 years. The literature reviewed was mainly from top journals, sourced from various databases, including ACS, ScienceDirect, SpringerLink, and Web of Science, and keywords like HBCD, air, soil, water, sediment, and biota were used. The objective of this study was to summarize the available information on HBCD and to discuss and compare the levels of HBCD in various environmental media in China until HBCD was banned in 2021. We provide a comprehensive overview of the spatial distribution of HBCD in these environmental media and have considered the causes of the pollution.

## 2. HBCD Distribution in Air

Long-range transport of HBCD would occur from contaminated sites to the atmosphere through volatilization or particles bound under certain environmental conditions [10,11]. Only a few studies were found on HBCD in the air in China and were mainly of Beijing and Guangdong Province (Table 1). The levels of HBCD in Beijing [12], Guangzhou [10], and Foshan [13] ranged from 0.69 to 1800 pg/m^3^, which were comparable with or slightly higher than those at the remote or urban sites in the United States (Michigan: 0.2–8.0 pg/m^3^, Louisiana: 0.2–6.2 pg/m^3^, Arkansas: 0.2–11 pg/m^3^, Chicago: 0.9–9.6 pg/m^3^) [14], but were comparable with or significantly lower than those in Europe (Stockholm: 76–610 pg/m^3^, Sweden: 1.07 × 10^6^ pg/m^3^, Norway: 0.2 × 10^6^–1.5 × 10^8^ pg/m^3^) [11,15,16]. This may be due to the fact that Europe was the first continent to use HBCD as a replacement for penta- and octa-BDE flame-retardant products and its consumption of HBCD accounted for more than half of the world market demand, according to a technical report.

Gas-particle distribution could affect the deposition, transport and subsequent fate of HBCD [10,12]. HBCD was primarily distributed in environmental solids, such as dust, and tended to deposit near point sources [13,17,18,19]. Yu [10] observed that level of HBCD in the particle phase was much higher than that in the gas phase at all four sampling sites (urban, city background, and industrial site) in Guangzhou. At national level, HBCD concentrations in outdoor dust in China were highly variable (0.133 to 25,400 ng/g). Similarly, in some cities, such as Shanghai and Beijing, the concentration values in outdoor dust also varied considerably, from 0.3 to 249 ng/g and 5.3 to 2580 ng/g, respectively [20]. In addition, HBCD concentration in outdoor dust was positively correlated with the dust particle size. This result was observed in Chongqing and Beijing [21,22].

Serious HBCD pollution was found in some areas affected by the HBCD sources. Very high concentrations of HBCD have been found in Zhejiang (12,400 ng/g), Tianjin (20,300 ng/g), and Guangdong (25,400 ng/g), which were more than 10–100 times those in dust from areas away from pollution [23]. This may be because the sampling points were located near EPS-production facilities and might be affected by the release from the production of HBCD-related products. Therefore, attention should be paid to the occupational exposure risk, contamination remediation, and environmental management in these places where HBCD was produced and used.

Because HBCD was mainly used in building materials and upholstery textiles as a flame retardant, urban areas were the main source of HBCD, as was reported in a study on the atmosphere of some cities in Southern China and in the BSEF technical report about HBCD applications [10,13,24]. Some indoor places like workplaces presented large quantities of HBCD-containing foams and electronic products, which could lead to high concentrations of HBCD in the air of the area due to volatilization or wear and tear of these products [13,22,25]. Total HBCD concentrations at workplaces (48.2 pg/m^3^) are higher than those in offices (8.21 pg/m^3^) and homes (5.43 pg/m^3^) in Guangzhou and Foshan [13]. HBCD released from urban sources was a substance in the air that was most likely to adversely affect humans. Many people spend most of their time indoors; therefore, the contribution of HBCD in indoor dust to human exposure was generally much higher than that outdoors. Therefore, the concentration of HBCD in indoor air deserves attention.

## 3. HBCD in Soil

### 3.1. Distribution and Levels of HBCD in soil

**Figure 1:**
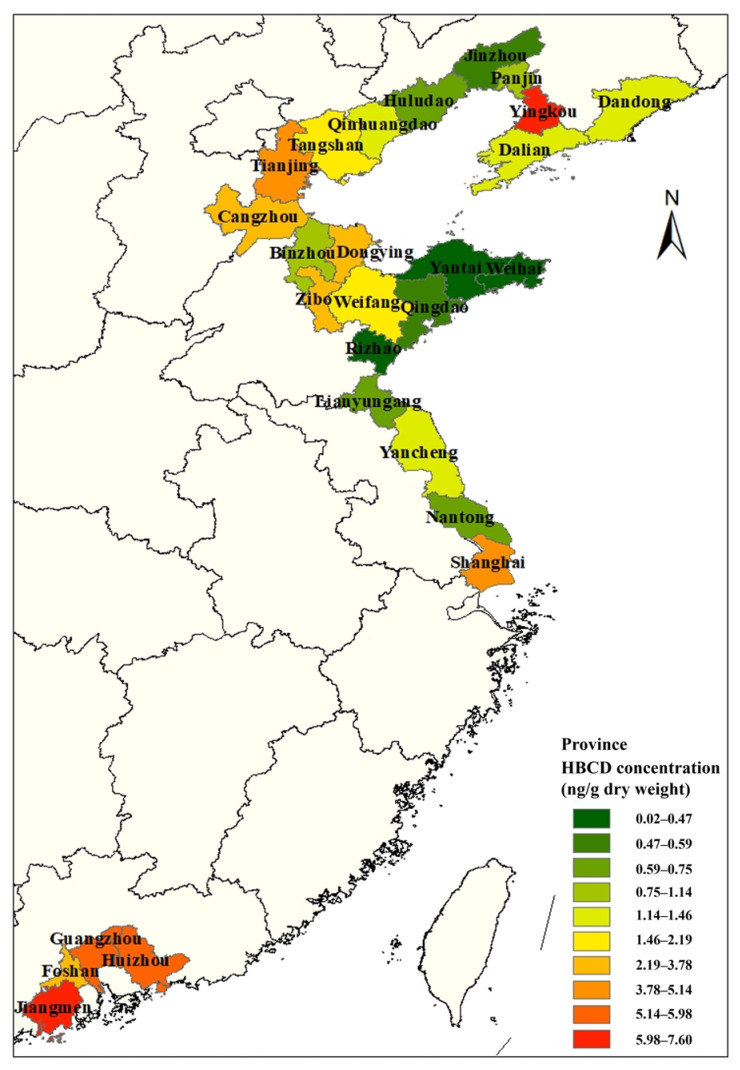
Distribution of HBCD concentration in soil in China.

Soil is a sink for HBCD, and there are many studies on HBCD in soil. Research from recent studies is collated in Figure 1 to illustrate the distribution of HBCD in soil in China. Most studies on HBCD in soil in China have been concentrated in the northeast coastal areas and, after that, the Pearl River Delta region. This may be due to the fact that the HBCD-related products in China are mainly produced in the coastal areas. HBCD showed regional distribution in soil, with varying concentrations from 0.88 to 23,200 ng/g across different areas [5,26,27]. Spatially distributed HBCD concentrations in soil decreased from Southeast to North China, except for Yingkou. For instance, HBCD concentration is higher in Jiangmen (7.6 ng/g) [28] compared to Weihai (0.449 ng/g) [6].

**Figure 2:**
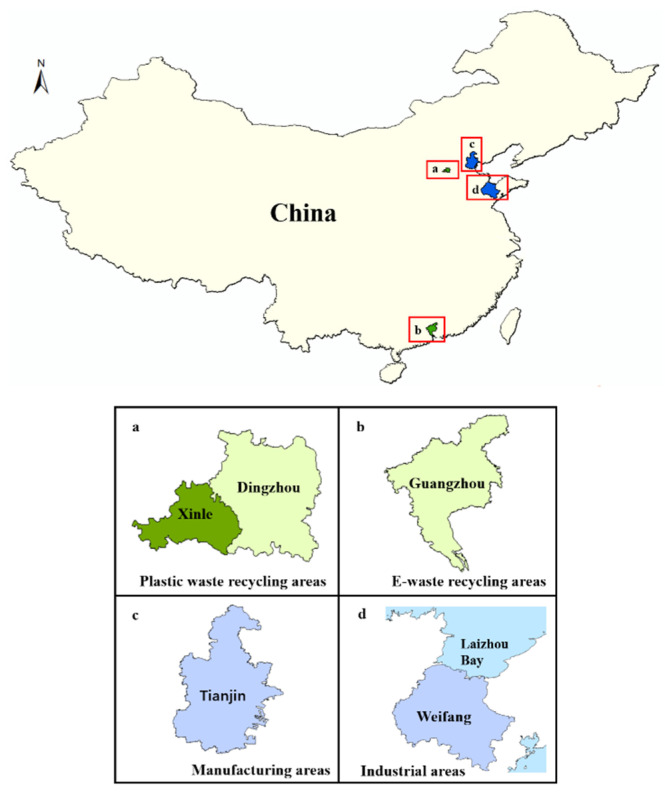
Different sources of HBCD in the surface soil.

HBCD in soil mainly comes from the local discharge of industrial wastewater and waste and/or deposition of atmospheric pollution from HBCD-related industrial processes. Generally, high concentrations of HBCD were found in soils near HBCD manufacturing plants, followed by soils near plants producing HBCD-related products (Figure 2) [26,27,29,30]. For example, the highest HBCD concentration measured in China (11,700 ng/g) was found in soil near one of the biggest HBCD manufacturing facilities in China [29]. Similarly, HBCD at concentrations of up to 6901 ng/g was detected in soil near HBCD manufacturing facilities in Laizhou Bay, East China [30]. However, the concentrations of HBCD detected in soil near the HBCD-related production and processing plants in Tianjin (1730 ng/g) and Weifang (560.4 ng/g) were lower compared to the above-mentioned areas [8,26]. Additionally, HBCD-related product wastes, including plastic waste, e-waste, and textiles, are also sources of HBCD pollution in the environment. For example, high concentrations of HBCD in soil samples near plastic-waste-treatment sites (11.0–624 ng/g) were detected in Dingzhou and Xinle [31]. Recycled plastic waste consisted mainly of EPX and XPS. E-waste and textile also contributed to increased HBCD concentrations in the environment; although, the amount of HBCD used in textiles and electrical/electronic equipment was relatively small. For example, HBCD concentrations in soils near the e-waste-recycling areas in Guangzhou were at moderate levels (0.38–284 ng/g), which was consistent with the level of HBCD detected in soil in Ningbo (below detection limits-102.6 ng/g), where the textile and electronic equipment industries are well developed.

Notable variations in HBCD concentrations exist across various soil types. For example, a study conducted in Ningbo analyzed six types of soil and revealed that significantly higher concentrations of HBCD were observed in waste-dumping sites (mean: 67.4 ng/g) and industrial areas (mean: 37.9 ng/g), followed by traffic areas (mean: 31.8 ng/g), residential areas (mean: 14.1 ng/g), vegetable soils (mean: 11.0 ng/g), and farmland soils (mean: 7.75 ng/g) [32]. Similarly, in Dingzhou and Xinle, the highest HBCD concentrations were observed in disposal/recycling sites (11–624 ng/g), followed by roadside soil (2.96–5.4 ng/g), and farmland soil (8.69–55.5 ng/g) [31]. However, only slight difference of HBCD concentration was observed in the different soil types in Chongming Island, where there is no industrial activity. The average concentration of HBCD in tideland soils (38.9 pg/g) was comparable to that in road soils (34.1 pg/g), and woodland soils (27.8 pg/g), and was only twice higher than in farmland soils (15.3 pg/g) and grassland soils (15.1 pg/g), respectively [33]. In addition, HBCD concentrations in soil clearly showed a decreasing trend with increasing distance from the point source of HBCD [6,8,11,30,34]. It was also found that within a certain distance from the point sources of HBCD, the concentration of HBCD in soil decreased dramatically with distance, but beyond that distance the decrease was slow. For example, centered on the biggest HBCD manufacturing plant in China, HBCD concentrations in soil decreased sharply by 11,600 ng/g from the center to 2 km. While from a range of 4.0 km to 6.1 km from the plant, the HBCD concentration detected in soil decreased slowly only by 3.8 ng/g [29]. At a distance of 4 km from another HBCD manufacturing plant in China, a significant decrease in the concentration of HBCD in the soil was observed as the distance increased. And, the concentrations became constant when the distance was >4 km [30]. Similar spatial distribution of HBCD around point sources was also observed in soil samples collected from e-waste-recycling areas in South China [34].

### 3.2. Diastereoisomer Profiles in Surface Soils

**Figure 3:**
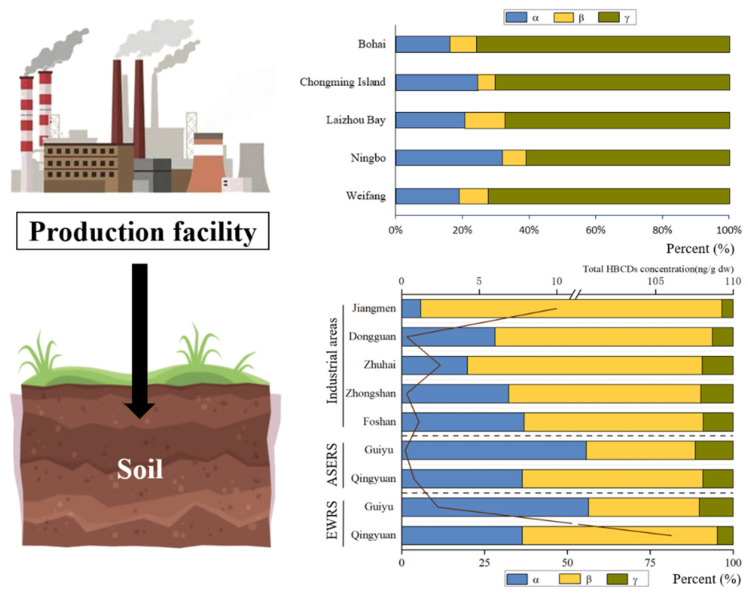
HBCD stereoisomer in soil in China (ASERS: areas surrounding the e-waste-recycling sites; EWRS: e-waste-recycling sites).

γ-HBCD usually shared the major proportion to the total HBCD in the soil, while certain cases revealed a relatively high proportion of α-HBCD (Figure 3). The composition of HBCD diastereoisomer in soils was strongly influenced by industrial emissions. For example, the diastereoisomer composition of HBCD in soils from the HBCD production facility area in China was found to be consistent with commercial HBCD products. Commercial HBCD consists mainly of 70–89% γ-HBCD, 10–13% α-HBCD, and 1–12% β-HBCD. The proportions to the total HBCD of γ-HBCD, α-HBCD, and β-HBCD were 76 ± 10%, 16 ± 8%, and 8 ± 6% in the soil near the largest HBCD manufacturer in China [29]. γ-HBCD was predominant (averaged 73.9%) in the brominated flame retardant’s production area in Shouguang, followed by α-HBCD (averaged 16.2%) and β-HBCD (averaged 9.9%) [6]. Similar patterns were also observed in soil samples collected from Laizhou Bay and Weifang [8,30]. And, in Guiyu, α-HBCD was the dominant isomer in soils from e-waste-recycling areas, accounting for 56–57% [34], which is similar to the profiles reported for soils near plastics’ industrial parks in Cangzhou (α-HBCD: 42.8–81.1%) [6]. The isomer pattern in HBCD-containing products and environment media can be changed by industrial processes, particularly extrusion molding and thermal cutting, before emission because of differences in the physical properties of the three isomers [6,29,34]. Lower γ-HBCD proportion (55.5%) was found in polystyrene hard plastics consumer products (general purpose polystyrene, high-impact polystyrene, etc.) with more industrial processing than EPS [35]. And, γ-HBCD was the predominant component of commercial technical HBCD products in EPS raw foam, but α-HBCD was found to be the main congener in particles emitted from thermal cutting of EPS. However, the diastereoisomer composition of HBCD in soils differed between point-source and non-point-source sites. For example, in Shouguang, the HBCD diastereoisomer composition in soils in point-source sites (65.3% γ-HBCD, 23.2% β-HBCD and 11.5% α-HBCD) showed significant differences from that in non-point-source sites (56.4% γ-HBCD, 23.8% α-HBCD and 19.7% β-HBCD) (*p* < 0.028) [36].

## 4. HBCD in Water, Sludge, and Sediment

### 4.1. HBCD in Water

#### 4.1.1. Distribution and Levels of HBCD in water

**Figure 4:**
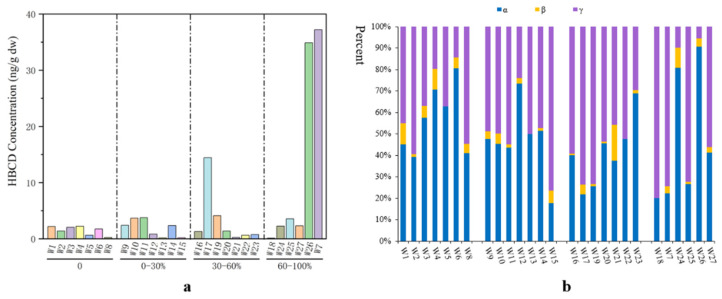
Concentration of HBCD (**a**) and corresponding isomers (**b**) in sludge from sewage-treatment plants in Shanghai.

Only a few studies on HBCD in water have been published in the past. There were three main sources of HBCD in natural water: discharge of industrial wastewater, direct discharge of domestic sewage, and atmospheric wet deposition. The concentration of HBCD in water bodies is both related to human activities, such as direct discharge of domestic sewage and effluent from sewage-treatment plants, and affected by its solubility (2.1–28.8 μg/L at room temperature) [29,37,38]. In natural rivers along the southern coast of the Bohai Sea, the concentrations of HBCD were relatively low, ranging from 4.98 to 41.3 ng/L [29]. Using liquid chromatography coupled to mass spectrometry with electrospray ionization, HBCD has been detected in Taihu Lake at concentrations ranging from not detected to 0.37 ng/L [38]. In water samples from the Yellow Sea, HBCD concentrations range from 0.12 to 2.23 ng/L. However, in many rivers receiving industrial wastewater, the concentration of HBCD has exceeded the range permitted by international standards (1.6–500 ng/L) [39]. For example, the HBCD concentration in wastewater discharged from the HBCD plant was 27.9–5080 ng/L, and the water samples with the highest concentration of HBCD (5080 ng/L) were collected near large HBCD producers on the southern coast of the Bohai Sea [29]. In urban environments, wastewater-treatment plants (WWTPs), and especially those treating industrial wastewater, are a crucial sink for HBCD [40]. The type of industrial wastewater influences the HBCD concentrations in WWTPs. For example, high HBCD concentrations (37.2 and 34.9 ng/g) have been found in WWTPs in Shanghai that mainly treat wastewater from motorcycle and automobile industries where HBCD is used (Figure 4a) [41].

#### 4.1.2. Diastereoisomer Profiles in Water

α-HBCD and γ-HBCD typically dominated in the water. For example, at the southern coast of the Bohai Sea, the concentration of α-HBCD was the highest (1.23–1800 ng/L), followed by γ-HBCD (1.10–2150 ng/L) and β-HBCD (0.85–1120 ng/L) [29]. Similar findings were observed in the Pearl River, with concentrations of 0.0075–0.0276 ng/L for α-HBCD, 0.0041–0.0545 ng/L for γ-HBCD, and 0.0018–0.0071 ng/L for β-HBCD. In addition, at the northern coast of the Yellow Sea, γ-HBCD is dominant, accounting for 73–100% [42,43].

### 4.2. HBCD in Sludge of Wastewater Treatment Plants

Due to its strong hydrophobicity (log*K*_ow_ of 5.4–5.8), HBCD has a propensity to adsorb onto suspended particulate matter and accumulate in sludge during wastewater treatment [44,45]. To date, there have been few studies on HBCD in sludge of wastewater-treatment plants in China. Two publications have reported on HBCD concentrations in sludge in Shanghai (0.1–37.2 ng/g) (Figure 4a) [41] and the Pearl Delta River (112–136 ng/g) [46].

A comparison with the 2001 national sewage sludge survey conducted by the U.S. Environmental Protection Agency reveals that sludge from Shanghai exhibits lower concentrations of HBCD (mean: 4.7 ng/g) than the global average (mean: 19.8 ng/g) [47]. In terms of annual production in China, the proportion of HBCD that ends up in sewage sludge is extremely low (<0.002%) [41]. The composition of HBCD congeners in sewage sludge in Shanghai is mainly dominated by α-HBCD or γ-HBCD (Figure 4b). α-HBCD was the most abundant in 10 sludge samples with mean proportions of 48.0%. γ-HBCD dominated in the other 17 sludge samples in Shanghai (mean: 47.9%), followed by α-HBCD and β-HBCD, which is similar to the composition of sewage sludge in the United States [47], and Spain (67.4% for γ-HBCD, 31.5% for α-HBCD, 1.1% forβ-HBCD) [48]. Previous studies have demonstrated that γ-HBCD exhibits the highest hydrophobicity among the three HBCD isomers, leading to its accumulation in solid matrices such as soils and sediments [33,34,49,50]. However, the isomer profiles of HBCD in Chinese sewage sludge have not been thoroughly investigated, necessitating further analysis.

In the Pearl River Delta region of China, HBCD concentrations (112–136 ng/g) in sewage-treatment plant sludge are much higher than those in river sediment from the same region (not detected to 31.6 ng/g) [46]. Similar results have been found in Spain [51] and Turkey [52]. This is mainly because the sludge from sewage-treatment plants has a high organic-matter content, and organic matter is a strong adsorbent of HBCD [53]. Consequently, HBCD remains in the sludge, resulting in a relatively high concentration. Therefore, the concentration of HBCD in sewage-treatment-plant sludge (13.1–616.2 ng/g) is generally 100–1000 times that in river sediment in the same area [51,52,54].

### 4.3. Occurrence and Distribution of HBCD in Sediment

Many studies have shown that because of its low water solubility, HBCD is readily adsorbed by particles in aquatic environments [11]. It then enters sediments through sedimentation and other processes, and sediments are one of the main storage sites of HBCD in the natural environment [50].

#### 4.3.1. Distribution and Levels of HBCD in sediment

**Figure 5:**
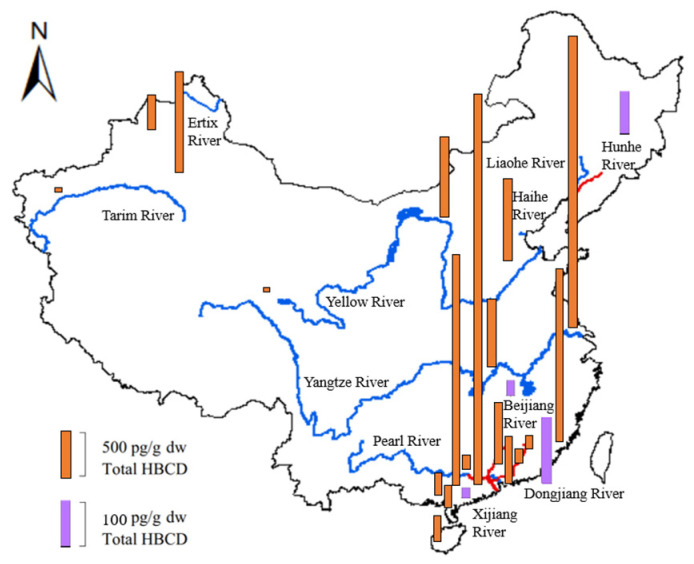
Geographical distribution and level of HBCD in sediments from several river drainage basins in China (blue lines indicate seven rivers, and red lines indicate four tributaries).

Figure 5 shows the geographical distributions and concentrations of total HBCD in sediments from the Yellow River, Yangtze River, Haihe River, Pearl River Delta, Liaohe River, Hunhe River, Ertix River, Tarim River, and some tributaries (e.g., the Dongjiang River, Beijiang River, and Xijiang River).

The HBCD concentrations measured in sediment from the seven rivers listed above range from not detected to 206 ng/g [50]. Generally, HBCD concentrations in sediment increased from the North to the Southeast of China (Figure 5). Compared with most other regions in the world, except for Europe and the coast of Korea [55], the Yangtze River had very high HBCD concentrations. The average HBCD concentration in sediments from the Yangtze River drainage basin was as high as 206 ng/g, which was much higher than that from the second-most polluted area, the Pearl River drainage basin (0.03–31.6 ng/g) [46]. HBCD concentrations in sediments from the remaining five rivers (Haihe River, Ertix River, Yellow River, Tarim River, and Liaohe River), which were all located in North China, were all lower than 0.5 ng/g. The HBCD concentrations decreased from the lower reaches to the upper reaches of the rivers. For example, the HBCD concentration in sediments from the upper reaches of the Hunhe River in Northeast China (0.05–1.42 ng/g) is relatively low compared with that from the lower reaches (0.10–25.8 ng/g) [56]. Similar spatial distributions of HBCD have been observed in sediments from the Yangtze River and Pearl River [50].

For tributaries of these rivers, the total HBCD was at a low level. The highest HBCD concentrations were detected in the Dongjiang River (mean: 6.89 ng/g) [46], followed by the Hunhe River (3.74 ng/g) [56], Zhujiang River (2.85 ng/g) [46], Shunde tributaries (1.43 ng/g) [46], Beijiang River (0.69 ng/g), Xijiang River (0.44 ng/g), and Dayanhe River (0.21 ng/g). The concentration of HBCD in sediments from Taihu Lake was basically at the same level, ranging from 0.168 to 2.66 ng/g dw, in 2009, and 0.046 ng/g dw to 2.56 ng/g dw, in 2010 [38,57].

Numerous industrial, commercial, and urban activities can affect the distribution of HBCD. The HBCD concentrations in sediments from industrial or urban areas were significantly higher (*p* < 0.023) than those from rural or less-industrialized areas. For instance, higher HBCD concentrations were found in sediments from the downriver section of the Hunhe River, which was exposed to industrial effluent from Shenyang and Fushun. Similar results have been found in the Pearl River and Yangtze River Delta, which are on the Southeast coast of China in regions with high levels of industrialization and high population densities [50]; the Kuzuryu River in Japan, which is severely polluted with effluent from the textile industries containing HBCD [58]; and Onsan Bay in Korea, which is a heavily industrialized and urbanized region [55]. The mean HBCD concentrations in the Beijiang River, Pearl River Delta, Xijiang River, and Dayanhe River were 0.69, 0.50, 0.44, and 0.21 ng/g, respectively [46]. The HBCD concentration in Laizhou Bay near manufacturing facilities ranged from 2.93 to 1029 ng/g [30]. Higher concentrations of HBCD have been observed in certain regions of China, likely due to intensive industrial activity, relatively high population density, and market demand for HBCD in these areas. These findings indicate that industrial activities and urbanization serve as potential sources of HBCD.

#### 4.3.2. Diastereoisomer Profiles in Sediment

Generally, γ-HBCD is the dominant isomer in sediment. This has been observed in sediment from the Hunhe River and coast of the northern Yellow Sea in China [56,59], lakes in England [60], the coast of Korea [55], and Tokyo Bay in Japan [61]. The contribution of γ-HBCD to the total HBCD concentration was consistent with the relative abundance (70–95%) of γ-HBCD in commercial technical products available in China, and with the concentrations measured in soils near manufacturing plants in North China [31]. For example, in Taihu Lake, γ-HBCD was the dominant isomer, accounting for 64.5–87.7% of total HBCD [57].

Numerous studies have reported variations in the isomer profiles of sediment samples. Specifically, certain studies have identified elevated concentrations of α-HBCD in sediment samples [62]. The proportions of HBCD isomers in different environmental matrices are potentially affected by thermal isomerization of HBCD-containing products [63], photolysis under natural light [59], and different degradation kinetics under aerobic and anaerobic conditions (α-HBCD slower than β- and γ-HBCD) [64,65].

## 5. HBCD in Biota

As a semi-volatile organic pollutant, the main ways for HBCD to enter organisms are through migration of soil and water pollution [31], respiratory intake [66], and enrichment via the food chain [3,67]. The concentrations of HBCD in organisms are affected by both its concentration in the environment [46,68] and the species [69].

### 5.1. HBCD in Animals

**Figure 6:**
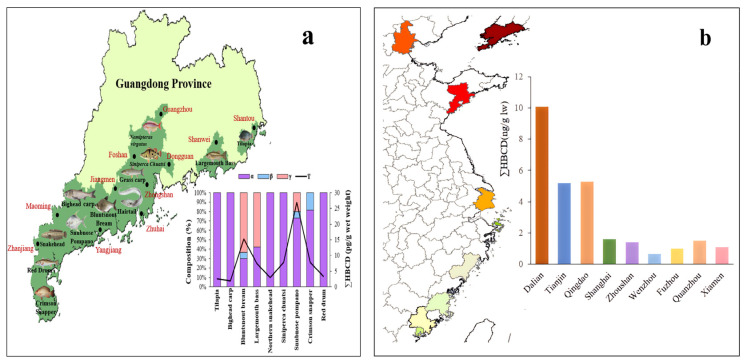
(**a**) Geographical distribution of HBCD in various types of aquatic biota in Guangdong Province and (**b**) concentration of HBCD in consumer fish in nine coastal cities (The color depth in the figure represents the concentration of HBCD in consumer fish).

The overall distribution of HBCD in various types of aquatic biota in China is shown in Figure 6. In many studies, fish, and shellfish are the common target aquatic species of HBCD, and the concentration of HBCD varies greatly among different aquatic species and regions. Because of the lipophilicity of HBCD, the content of HBCD in organisms is positively correlated with its fat content. This conclusion is proved by the distribution in fish tissues, and studies have found that the concentrations of HBCD in fish tissues decreased in the order of eggs > liver > muscles [70,71]. HBCD concentrations in liver and eggs were higher than those in muscles of freshwater fish from the Yangtze River [72].

Overall, HBCD concentrations in aquatic biota in China are relatively low. HBCD concentrations in large yellow croaker and silver pomfrets, which are common consumer fish in coastal regions, from nine coastal cities (Shanghai, Zhoushan, Fuzhou, Dalian, Tianjin, Qingdao, Wenzhou, Quanzhou, and Xiamen) ranged from 0.62 to 8.7 ng/g lw (lipid weight) and from 0.57 to 10.1 ng/g lw (lipid weight), respectively [73]. The HBCD concentration of bluntsnout bream (*Megalobrama amblycephala*) and snubnose pompano (*Trachinotus blochii*) in Guangdong was 0.471–0.665 ng/g lw [74]. Interestingly, the HBCD concentration increases from the south (average: 1.2 ng/g lw) to the north (average: 6.5 ng/g lw) in silver pomfrets (Figure 6b) and other aquatic species, which may be due to the fact that the consumption of EPS/XPS containing HBCD is less in the south than in the north. HBCD concentrations (not detected–0.194 ng/g lw) have been detected in 12 consumer fish species in 11 coastal cities in South China (Figure 6a) [74]. Those in apple snails (*Ampullaria gigas* spix) and grass carp (*Ctenopharyngodon idellus*) from East China are 3.5–6.55 ng/g lw and 14.9–67.8 ng/g lw, respectively [67].

The obtained results demonstrate similarity to the concentrations found in benthic shellfish, including oysters, scallops, and mussels, in the northern region of the Yellow Sea, which ranged from 0.87 ng/g to 67.08 ng/g [59]. However, they appear slightly lower in comparison to the concentrations detected in mollusks from the Bohai Sea, which ranged from 25.1 ng/g to 148.87 ng/g lw [75].

The HBCD-related industrial activities likely affect the concentration and geographical distribution of HBCD in biota. High concentration of HBCD is detected in rivers and lakes related to HBCD pollution in China. For instance, the high concentrations of HBCD in aquatic species (goby, silver carp, loach, and freshwater shrimp) (7.09–815 ng/g lw) was found near manufacturing facilities in Laizhou Bay which was the important HBCD production base in China [30]. In the e-waste-recycling area of China, HBCD concentrations of up to 186, 377, and 1791 ng/g lw have been found in crucian, loach, carp, and winkle, respectively [76]. HBCD concentrations in freshwater fish from the low reach of the Yangtze River ranged from 11 to 330 ng/g lw, where large amount of polymer raw materials, textiles, electronic appliances, and fine petrochemical plants are located [72]. HBCD concentrations were detected in rats (mean: 40.3 ng/g lw, range: 22.1–51.1 ng/g lw) from an e-waste-dismantling region in East China [67]. HBCD has been detected in samples collected near an EPS material-manufacturing plant in Tianjin. In marine species such as shrimp, crab, and fish, the total concentration of HBCD ranged from 0.878 ng/g to 44.8 ng/g [26]. Geographically, among the nine Chinese coastal cities, the highest HBCD concentrations were found in Dalian (large yellow croaker:5.2 ng/g lw; silver pomfrets:10.1 ng/g lw), which is located near Laizhou Bay in the Bohai Sea and has been seriously polluted by domestic and industrial effluent [73]. HBCD concentrations were observed to be higher in terrestrial passerine birds inhabiting urban and e-waste sites in the Pearl River Delta, while rural sites exhibited the lowest concentrations [77]. These results indicated that industrial activities related to HBCD affected the concentration level and distribution of HBCD.

### 5.2. HBCD in Plants and Mangrove Wetlands

The concentrations of HBCD decreased in the order of industrial areas > commercial areas > residential areas in tree bark [12,78]. These results are in the agreement with those from a study on tree bark from 12 locations around the world [79]. In Xinle and Dingzhou, which are major plastic-waste-recycling areas in North China, HBCD concentrations ranged from 3.47 ng/g in spinach to 23.4 ng/g in leeks [31]. High concentration of HBCD was detected in holly, cypress, and pine (3.45–2494 ng/g dw) collected around an EPS manufacturing plant in Tianjin [26]. Surprisingly, the highest HBCD have been detected at concentrations ranging from 8.88 to 160,241 ng/g dw in plants (cypress, reed, and seepweed) near HBCD manufacturing facilities in Laizhou Bay [30]. These results showed that point sources of HBCD can have a wide impact on the concentration of HBCD of the plants. In addition, enrichment of HBCD in different tissues from terrestrial plants showed some regularity. HBCD concentrations decreased in the order of leaf wax > inner leaf > branch > bark [26].

In coastal intertidal areas, mangrove wetlands are unique ecosystems that are natural sinks for many pollutants. In the northeastern coast of Shenzhen Bay, South China, the concentrations of total HBCD in mangrove plants ranged from 0.016 to 194 ng/g dw, in which the average concentrations of total HBCD in stems, roots, and leaves were 766, 329, and 298 pg/g dw. The total concentrations in this region were relatively low compared to other regions worldwide. Mangrove plants exhibit tissue-specific accumulation of HBCD. γ-HBCD was the primary isomer found in the roots, while α-HBCD dominated in leaves and stems. Furthermore, α-HBCD was the dominant isomer in aboveground tissues, potentially due to metabolism, isomer-specific translocation, and/or isomerization in mangrove plants. Enantioselective enrichment of (−)-α-, (−)-β-, and (+)-γ-HBCD was observed in all mangrove plant tissues, suggesting that the tissues exhibit enantioselectivity for HBCD. The logarithms of translocation factors for HBCD isomers and logKow exhibited a negative correlation (*p* = 0.03), indicating that passive translocation of HBCD is driven by moisture movement during transpiration [66].

### 5.3. HBCD in Human Breast Milk

The analysis of HBCD in human breast milk suggested that global exposure of humans to HBCD was low. As China was a country with high production and use of HBCD, increasing HBCD concentrations in breast milk were detected in recent years in some studies. According to data from two national breast milk surveys on HBCD, the concentration of HBCD in Chinese people has increased approximately six times from 0.944 ng/g lw in 2007 [80] to 6.83 ng/g lw in 2011 [81]. In Beijing, the HBCD concentration in breast milk in 2014 (mean: 5.67 ng/g lw) [82] was twice higher than those measured in 2011 (mean: 2.4 ng/g lw) [83]. HBCD is mainly used as a flame retardant for thermal insulation materials (such as polystyrene foams) and textiles which are used in residential and commercial upholstered furniture. Compared with the northern region, the HBCD concentration in breast milk was relatively low in South China. For example, the mean concentration of HBCD in breast milk was 1.42 ng/g lw in Shanghai [84] and 1.82 ng/g lw in Shenzhen [85], which was lower than that in Weifang, North China (mean: 2.86 ng/g lw) [86]. This may be due to the heating in winter in northern China and the fact that most HBCD manufacturers are located in northern China, which makes HBCD more widely used in North China. In addition, since controls of HBCD were implemented by the European Union in 2013, HBCD concentrations in breast milk in Britain [87,88] and France [89,90] have decreased. Therefore, in China, the content of HBCD in breast milk is expected to decrease since the production and use of HBCD was completely banned in December 2021.

### 5.4. Diastereoisomer Profiles in Biota

Generally, the dominant isomer is α-HBCD in most biota, including plants, river fish, aquatic invertebrates, marine mammals, birds, and rats [31,67,71,72,73,91]. This is followed by γ- and β-HBCD. Compared with sediments, where γ-HBCD is dominant, the dominance of α-HBCD in biota indicated that the bioaccumulative potential of α-HBCD was higher than that of γ-HBCD. This is likely due to the faster metabolism of γ-HBCD compared to α-HBCD, leading to the enrichment of α-HBCD. This pattern of dominance agrees with research on juvenile rainbow trout and harbor seals [92]. In the Yangtze River, α-HBCD is dominant in different tissues from freshwater fish, including the liver (80%), muscles (60%), and eggs (40%) [72]. These results agree with those from a study on fish from other areas like the North Sea estuaries, Europe [93]. In East China, α-HBCD is the dominant isomer in grass carp (89.8%), frogs (55.8%), and apple snails (44.9%) [67]. However, in grasshoppers (65.8%) and dragonflies (73.6%), γ-HBCD is dominant, which is consistent with the profiles in spotted doves (72%) and Chinese francolin (63%) from an e-waste-recycling region in South China [94]. Therefore, these species might be exposed to nearby sources of HBCD pollution.

HBCD isomers exhibited tissue-specific bioaccumulation patterns. For instance, in crabs, α-HBCD exhibited preferential accumulation in the spermary and ovary, whereas β-HBCD and γ-HBCD were found to accumulate in muscle tissues. A similar trend was observed in the roe and muscles of goby fish [30,31]. HBCD in plants also exhibits high enantioselectivity [31]. For example, the percentage contributions of α-, β-, and γ-HBCD in spinach root and garlic root collected from Yihezhuang were 48.5%, 24.4%, 27.1%, as well as 56.9%, 29.4%, and 13.8%, respectively.

To study the cumulative effect of HBCD in biota, researchers have used the trophic amplification factor (TMF). The concentration level of HBCD may affect its cumulative effect in biota. For example, in aquatic food webs in areas with high HBCD concentrations, the TMF is greater than one [26,67], suggesting a trophic magnification. While in aquatic food webs in areas with low HBCD concentrations, such as the Tibet Plateau and Singapore, the TMF is one, which shows that some trophic dilution occurs [95,96]. Similar results have been found in a terrestrial food web [67].

## 6. Conclusions

In all environmental media, relatively high HBCD concentrations have been found in HBCD-polluted areas. Because of its relatively high volatility and hydrophobicity, HBCD is mainly distributed in atmospheric particles (dust) rather than in the gas phase. The HBCD concentration ranges in outdoor dust and outdoor air in some areas were 0.133 to 25,400 ng/g and 3.09 to 1800 pg/m^3^, respectively. Furthermore, there is a positive correlation between HBCD concentrations and total suspended particulate matter in the atmosphere (*R*^2^ = 0.531, *p* < 0.01). In soil, the total HBCD concentration ranges from 0.88 to 23,200 ng/g and decreases from the Southeast to the North. In rivers, the HBCD concentration in water was between 4.98 and 41.3 ng/L, while the concentrations of HBCD in sediment was from not detected to 206 ng/g and decreased from the southeast to the north of China. In aquatic biota, HBCD concentrations in the Bohai Sea and Yangtze River were the highest in China. For terrestrial species, HBCD concentrations were linked to the pollution of HBCD and the function of each district. Enrichment of HBCD in different tissues from terrestrial biota showed some regularity. In breast milk, the total HBCD concentration increased year by year, from 0.944 ng/g lw in 2007 to 6.83 ng/g lw in 2011. However, the concentration of HBCD was expected to decline in the future after China completely prohibited the production and use of HBCD in 2021. Studies have shown that γ-HBCD is the dominant isomer in soil, water, and sediment, while in biota, α-HBCD is dominant. Further studies are needed to investigate the mechanism behind HBCD isomer dominance and to identify factors influencing the isomer profile.

## Figures and Tables

**Table 1:** Concentrations of HBCD in air from various sites.

Location	Type	Total HBCD (Mean, Range) pg/m^3^	Method	Reference
Beijing	urban	390, 20–1800	UPLC-MS/MS	[12]
Guangzhou	industrial site	0.7, 0.3–1.2	LC-MS/MS	[10]
Guangzhou	industrial site	0.9, 0.4–1.8	LC-MS/MS	[10]
Guangzhou	urban	3.1, 2.2–3.9	LC-MS/MS	[10]
Guangzhou	city background	1.7, 1.1–2.3	LC-MS/MS	[10]
Michigan	remote	1.2, 0.2–8.0	HPLC-MS/MS	[14]
Louisiana	remote	0.6, 0.2–6.2	HPLC-MS/MS	[14]
Arkansas	semirural	1.6, 0.2–11	HPLC-MS/MS	[14]
Chicago	urban	4.5, 0.9–9.6	HPLC-MS/MS	[14]
Sweden	producing XPS	1.07 × 10^6^	GC-MS	[11]
Norway	producing XPS	12.2 × 10^6^, 0.2 × 10^6^–1.5 × 10^8^	GC-MS	[15]
